# Evaluation of Urine Exosome Lecithin Cholesterol Acyltransferase as a Biomarker for Diabetes Diagnosis and Dyslipidemia

**DOI:** 10.1002/dmrr.70133

**Published:** 2026-03-03

**Authors:** Tianci Liu, Na Liu, Qian Meng, Tao Li, Man Zhang

**Affiliations:** ^1^ Clinical Laboratory Medicine Beijing Shijitan Hospital, Capital Medical University Beijing China; ^2^ Beijing Key Laboratory of Urinary Cellular Molecular Diagnostics Beijing China; ^3^ Institute of Regenerative Medicine and Laboratory Technology Innovation Qingdao University Qingdao China

**Keywords:** animal model, biomarkers, cell model, diabetes mellitus, LCAT, lipid metabolism

## Abstract

**Aims:**

Given the necessity for diabetes monitoring, investigating the expression of lipid metabolism‐related proteins may provide valuable insights for disease surveillance and mechanistic elucidation.

**Materials and Methods:**

LC‐MS/MS was used to analyse the expression of lipid metabolism‐related proteins in urine exosomes. Biomarkers were screened and their clinical significance was evaluated. Insulin‐resistant hepatocytes and diabetic mouse models were established to validate and explore the underlying mechanisms.

**Results:**

Logistic regression analysis revealed that elevated triglyceride (TG) and reduced high‐density lipoprotein cholesterol (HDL‐C) were independent risk factors for diabetes. In normal controls and those with prediabetes, females had higher total cholesterol (TC) and HDL‐C but lower TG compared to males, whereas no gender differences were observed in the diabetic group. 29 lipid metabolism‐related proteins were screened by proteomics, among which lecithin cholesterol acyltransferase (LCAT) was the target protein. Disordered lipid metabolism and upregulation of LCAT were observed in diabetic patients, insulin‐resistant cells, and diabetic mice. The liver may be a key organ of early diabetic injury, as evidenced by disorganised hepatocyte arrangement and compensatory LCAT production during the prediabetic stage. As the disease progresses to diabetes, hepatic steatosis significantly worsens, accompanied by elevation of serum and urine LCAT. Urine exosome LCAT not only holds diagnostic value but also exhibits distinctive dynamic changes: an early rise in diabetes followed by a decline during periods of poor glycaemic control (HbA1c ≥ 8% or FBG > 7.8 mmol/L).

**Conclusions:**

LCAT, a critical regulator of lipid metabolism, could serve as a novel biomarker for detection and monitoring of diabetes.

## Graphical abstract

Integrated proteomic and model studies reveal lipid metabolism dysregulation in diabetes, detectable even in prediabetes. LCAT, a key regulator, shows compensatory upregulation during progression. The characteristic dynamics of urine exosome LCAT provide a basis for its use as a non‐invasive biomarker for early diabetic detection and disease monitoring.

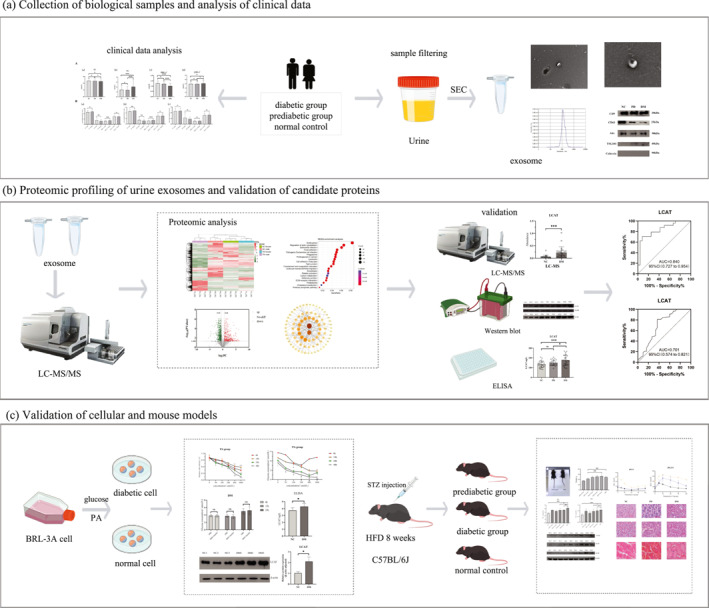

## 1 Introduction

Diabetes mellitus (DM) is a metabolic disorder characterised by chronic hyperglycemia, primarily resulting from defects in insulin secretion or impaired insulin action [1]. As of 2021, approximately 537 million individuals worldwide were living with diabetes, and this number is projected to increase to 783 million by 2045 [2].

Diabetes mellitus remains incurable to date [3]. Current clinical management of the disease primarily relies on repeated venous blood sampling for monitoring key indicators such as HbA1c and fasting blood glucose [4], but the long‐term invasive operation leads to a reduction in patient compliance. The study of non‐invasive diagnostic and monitoring markers has become an urgent research direction in the field of diabetes management.

As a noninvasive and easily accessible sample, urine is an attractive source of markers in large‐scale disease screening [5]. Exosomes are 30–150 nm vesicles secreted by cells [6]. They are widely present in body fluids, and their contents (such as proteins, miRNAs, and metabolites) exhibit significant differences under physiological and pathological conditions, providing a rich molecular library for the discovery of disease biomarkers [7, 8, 9, 10]. Therefore, urine exosomes are considered promising candidates for diabetes monitoring.

Studies have found a close interaction between lipid metabolism and diabetes, particularly type 2 diabetes [10]. Decreased HDL is not only an independent risk factor for T2DM but also interferes with the disease process through multiple mechanisms. Research has shown that HDL reduces the accumulation of lipotoxic substances in *β*‐cells by promoting reverse cholesterol transport and activates the PI3K/Akt signalling pathway to inhibit endoplasmic reticulum stress and mitochondrial apoptosis, thereby maintaining insulin secretion [11]. In addition, HDL enhances the membrane translocation of GLUT4 in skeletal muscle cells, improves the efficiency of glucose uptake, and inhibits the release of inflammatory factors from adipose tissue, thereby improving insulin resistance [11, 12].

In this study, proteomic technology was used to analyse the expression of lipid metabolism‐related proteins in urine exosomes of diabetic patients and to screen for candidate protein markers. The target protein was validated at the cellular and tissue levels by establishing a hepatocyte insulin resistance model and a diabetic mouse model, thereby providing novel biomarkers for the non‐invasive monitoring of diabetes and laying a theoretical foundation for investigating the mechanism of lipid metabolism disorders in diabetes (The experimental route can be found in the ‘Graphical Abstract’ [13]).

## 2 Materials and Methods

### 2.1 Patients

Patients with type 2 diabetes mellitus and prediabetes mellitus who visited Beijing Shijitan Hospital were enroled, and healthy people were selected as the control group according to the principle of age and gender matching. Written informed consent was obtained from all participants prior to the study. This study was approved by the Ethics Committee of Beijing Shijitan Hospital (sjtkyll‐lx‐2022‐134).

### 2.2 Cell Culture

Rat hepatocyte cell line BRL‐3A cells (Pricella Biotechnology Co. Ltd., Wuhan, China, product number: CL‐0036) were used to establish insulin resistance cell model by palmitic acid (PA) combined with high glucose stimulation. All cell culture processes were carried out in a 37°C constant temperature incubator with 5% CO_2_ and 95% humidity.

### 2.3 Mice

Eighteen 6‐week‐old SPF male C57BL/6J mice (SiPeiFu, Beijing, China) were divided into three groups: diabetic group (DM), prediabetic group (PD) and normal control group (NC). This experiment protocol was approved by the Experimental Animal Ethics Committee of Beijing Shijitan Hospital, Capital Medical University (sjtkyll‐lx‐2023‐073).

### 2.4 Exosome Isolation

In this study, urine exosomes were isolated using differential centrifugation combined with size exclusion chromatography (SEC) [14, 15]. Detailed methods are provided in the Supporting Information S1.

### 2.5 Mass Spectrometry Detection

Each sample was dissolved in 100 μL mobile phase A, centrifuged at 14000 g for 20 min, and the supernatant was taken and processed using high performance liquid chromatography. The dry peptide powder was dissolved in 0.1% FA water and the iRT standard peptide was added. Then, 1 μg was taken for mass spectrometry analysis. The ORBITRAP ECLIPSE mass spectrometer was used for data acquisition using DIA mode. The results of the mass spectra were interrogated in the Homo sapiens database within Uniprot (www.Uniprot.org) using the Spectronaut software. At the protein level, a 1% FDR was used as a filter, and each protein contained at least one unique peptide. Fold changes > 1.5 and *p* values < 0.05 were considered significant differences.

### 2.6 Western Blot

Samples were loaded onto 15% Tris‐HCl SDS‐polyacrylamide gels and transferred to PVDF membranes, followed by addition of blocking solution and shaking for 2 h. The primary antibodies were diluted at a ratio of 1:2000, followed by incubation at 4°C. After washing three times with TBST (15 min each), HRP‐labelled secondary antibodies were incubated for 1.5 h and washed with TBST. Subsequently, enhanced chemiluminescence was used for detection.

### 2.7 Enzyme‐Linked Immunosorbent Assay

The protein concentration of the samples was determined using the BCA method. After that, the concentration of the target protein was measured using ELISA kit of FineTest (Wuhan, China).

### 2.8 Determination of the Optimal Concentration and Action Time of Palmitic Acid and Glucose

200 μL of BRL‐3A cell culture medium was added to the control group, 200 μL of palmitic acid solution was added to the PA group, and 200 μL of ethanol was added to the PA control group, and then incubated in a carbon dioxide incubator for 6 h, 12 h, 24 h, and 48 h, respectively. Then, the CCK8 method was used to detect cell viability and calculate the glucose consumption of each group. The same method was used to determine the optimal concentration and action time of glucose.

### 2.9 Insulin Stimulation Test

BRL‐3A cell culture medium (200 μL) was added to the blank well group and the normal control group. BRL‐3A cells in the diabetes group were treated with appropriate concentrations of PA and glucose, and then incubated in a carbon dioxide incubator for appropriate time. After completion of the culture, the cells were washed twice with PBS solution, and 200 μL of insulin was added to each well to continue the culture for 30 min. The supernatant glucose concentration of each well was measured.

### 2.10 Establishment of Mouse Model of Type 2 Diabetes

Normal control mice were fed a standard diet, while prediabetic and diabetic groups were fed a high‐fat diet for 8 weeks. The diabetic group then received STZ injections, whereas the control and prediabetic mice received sodium citrate buffer for 5 days. After 1 week, blood glucose levels were measured, followed by an intraepithelial glucose tolerance test (IPGTT) and an intraepithelial insulin tolerance test (IPITT).

### 2.11 Haematoxylin‐Eosin Staining (HE)

Tissue sections were deparaffinised in xylene and rehydrated using a graded ethanol series. Haematoxylin staining was then applied for 10 min. Differentiation was carried out by applying a differentiating solution for 30 s followed by another distilled water rinse. Eosin staining was performed for 2 min, and excess dye was carefully removed. Dehydration was achieved through a reverse ethanol gradient, and the sections were subsequently cleared in xylene.

### 2.12 Immunohistochemical Staining

After deparaffinisation and rehydration, antigen retrieval was performed for 10–20 min. The slides were then cooled, washed with PBS, and treated with a 3% hydrogen peroxide solution for 10 min. Blocking was done for 60 min, followed by primary antibody incubation at 4°C overnight. After rewarming for 60 min at room temperature and washing with PBS, a HRP‐conjugated secondary antibody was applied. DAB substrate was added for 15 min in dark with microscopic monitoring. Finally, the sections were counterstained with haematoxylin, dehydrated and cleared in xylene.

### 2.13 Statistics

SPSS 19.0 and GraphPad Prism 9.0.2 software were used for statistical analysis. Normally distributed data are expressed as mean ± standard deviation (SD), non‐normally data as median and interquartile range (IQR). The diagnostic performance of the target proteins was evaluated using receiver operating characteristic (ROC) curve analysis.

## 3 Results

### 3.1 Part I: Clinical and Laboratory Characterisation of Diabetic Patients

#### 3.1.1 Clinical Characteristics

This study enroled three groups of participants: DM (*n* = 137), PD (*n* = 225), and NC (*n* = 261). No significant differences were observed in age or gender distribution across the three groups. The clinical characteristics of each group are presented in Supporting Information S2.

#### 3.1.2 Logistic Regression Analysis of the Incidence of Diabetes

Potential risk factors for diabetes were evaluated using univariate and multivariate logistic regression analyses. Multivariate analysis identified ALB, Urea, TG, HDL‐C, WBC, and MO% as independent risk factors for diabetes. Supporting Information S3 presents complete logistic regression results.

#### 3.1.3 Analysis of Changes in Clinical Lipid Metabolism Indicators

Compared with the NC and PD, diabetic patients exhibited elevated TG levels and reduced HDL‐C levels, with no change in TC or LDL‐C (Figure S1A). No sex‐based differences were observed in diabetic group, but females in NC and PD exhibited higher TC and HDL‐C and lower TG levels than males (Figure S1B).

#### 3.1.4 Identification of Urine Exosomes

**FIGURE 1:**
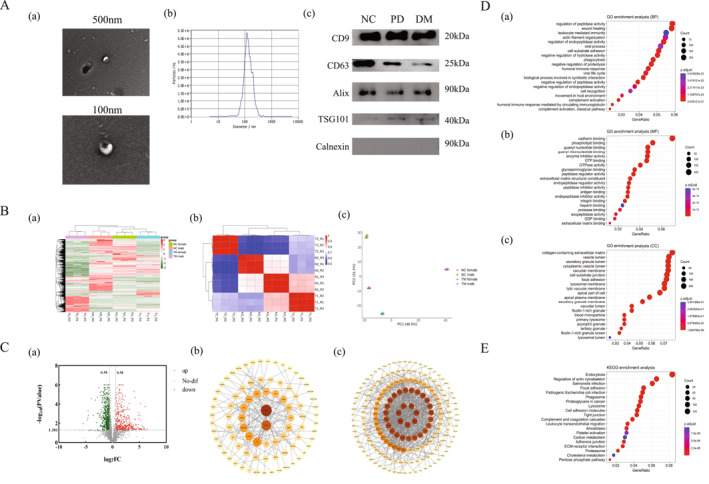
Proteomic analysis of urine exosomes from diabetic patients (A): Identification of urine exosomes from subjects. (a): Transmission electron microscope (TEM), scale bars 500 and 100 nm; (b): NTA; (c): Western blot, detection of exosome markers CD9, CD63, Alix, TSG101, and Calnexin. (B): Overall analysis of urine exosome proteomics of the subjects. (a): Cluster heatmap of urine exosome proteins in normal control and diabetic groups; (b): Heat map of correlation analysis of urine exosome samples in normal control group and diabetic group; (c): PCA plot of urine exosome samples from the normal control and diabetic groups. (C): Differential expression analysis of urine exosome proteins in diabetic patients. (a): Volcano plot of urine exosome protein expression in normal control group and diabetic group, red represents up‐regulated protein in diabetes, green represents down‐regulated protein; (b): PPI analysis of up‐regulated proteins expressed in urine exosomes from diabetic patients. The size of dots reflects the frequency or strength of the protein involved in the interaction; (c): PPI analysis of down‐regulated proteins expressed in urine exosomes from diabetic patients. The size of the dots reflects the frequency or strength of the protein involved in the interaction. (D): GO functional enrichment analysis of urine exosome proteins. (a): biological process (BP); (b): molecular function (MF); (c): cellular component (CC). The vertical axis shows significantly enriched pathways, the horizontal axis shows the proportion of proteins, the size of the dot indicates the number of genes, and the colour of the dot indicates the magnitude of the *p*‐value. (E): KEGG enrichment analysis of urine exosome proteins. The vertical axis shows significantly enriched pathways, the horizontal axis shows the proportion of proteins, the size of the dot indicates the number of genes, and the colour of the dot indicates the magnitude of the *p*‐value.

Urine exosomes were characterised in three aspects (Figure 1A). Under transmission electron microscopy (TEM), distinct exosome bilayer membrane structures were observed. Nanoparticle tracking analysis (NTA) revealed that the isolated urine exosomes had an average particle size of 141.4 nm. Western blot analysis demonstrated significant expression of CD9, CD63, Alix, and TSG101, while the negative marker Calnexin showed no detectable expression.

#### 3.1.5 Proteomic Analysis of Urine Exosomes

2970 proteins were identified by mass spectrometry. As shown in Figure 1B, the cluster heatmap showed that the MS data of DM and NC groups were stable (a) with strong intergroup correlation (b). Principal component analysis (PCA) further confirmed the reproducibility of the group and the significant intergroup differences (c). Compared with the NC group, 383 proteins were up‐regulated and 627 proteins were down‐regulated in the diabetic group, with strong PPI interaction (Figure 1C).

#### 3.1.6 Biological Function Analysis of Proteins

Gene Ontology (GO) enrichment analysis (Figure 1D) revealed that biological processes (BP) were primarily enriched in the regulation of peptidase activity, wound healing, and leucocyte mediated immunity. Molecular functions (MF) were predominantly associated with cadherin binding, phospholipid binding, and guanyl nucleotide binding. Cellular components (CC) were mainly represented by collagen‐containing extracellular matrix, vesicle lumen, and secretory granule lumen. KEGG pathway analysis identified significant enrichment in endocytosis and regulation of actin cytoskeleton, among others, with cholesterol metabolism ranking as one of the top metabolic pathways (Figure 1E).

#### 3.1.7 Global Analysis of Proteins Involved in Cholesterol Metabolism in Urine Exosomes

**FIGURE 2:**
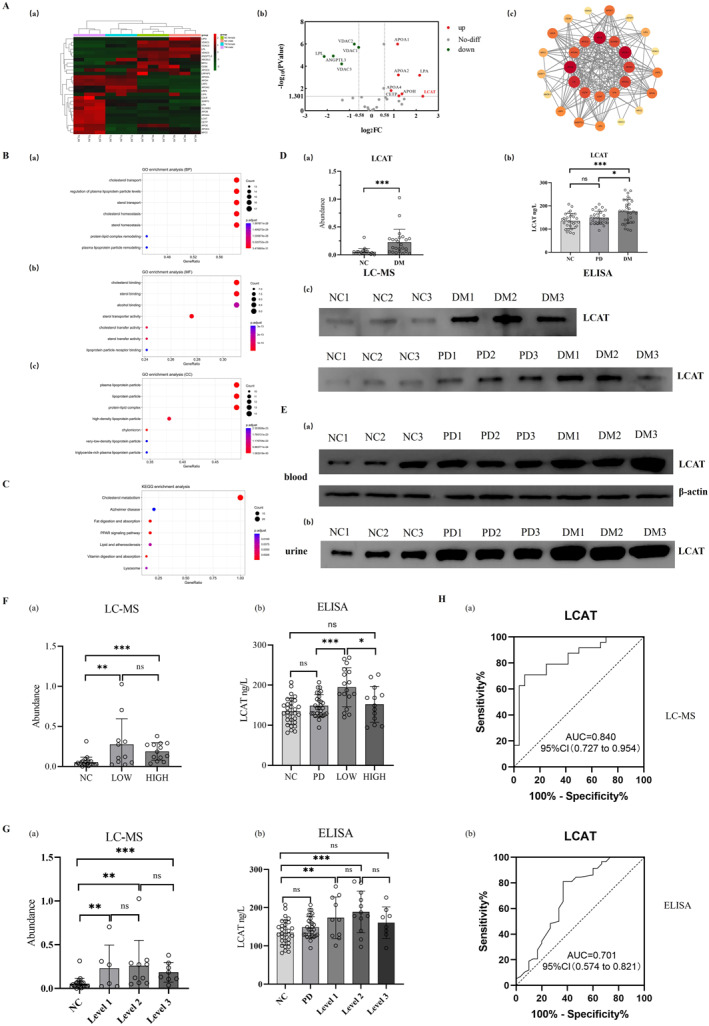
Screening and validation of proteins associated with cholesterol metabolism pathway in diabetic urine exosomes. (A): Overall analysis of urine exosome cholesterol‐related proteins. (a): Cluster heatmap of normal control group and diabetic group; (b): Volcano plot of protein expression in normal control group and diabetic group, red represents up‐regulated protein and green represents down‐regulated protein in diabetes group. (c): PPI of urine exosome cholesterol metabolism‐related proteins. The size of the dots reflects the frequency or strength of the interaction in which the protein is involved. (B): GO functional enrichment analysis of urine exosome cholesterol metabolism‐related proteins. (a): biological process (BP); (b): molecular function (MF); (c): cellular component (CC). The vertical axis shows significantly enriched pathways, the horizontal axis shows the proportion of proteins, the size of the dot indicates the number of genes, and the colour of the dot indicates the magnitude of the *p*‐value. (C): KEGG enrichment of urine exosome cholesterol metabolism‐related proteins. The vertical axis shows significantly enriched pathways, the horizontal axis shows the proportion of proteins, the size of the dot indicates the number of genes, and the colour of the dot indicates the magnitude of the *p*‐value. (D): Changes in the expression of cholesterol metabolism‐related target protein LCAT in urine exosomes. (a): Expression of NC group and DM group in mass spectrometry experiment; (b): Expression of NC group, PD group and DM group in ELISA assay; (c): Expression of the three groups of subjects in Western blot experiments. ns: no significance; *: *p* < 0.05; ***: *p* < 0.001. (E): Expression changes of cholesterol metabolism‐related target protein LCAT in blood and urine. (a): The expression of LCAT in the blood of NC, PD and DM groups, and *β*‐actin was used as the reference protein; (b): Urine LCAT expression in the NC, PD, and DM groups. (F): Analysis of urine exosome protein LCAT expression in diabetic patients with different HbA1c levels. (a): Expression of NC group, LOW group and HIGH group in mass spectrometry experiment; (b): Expression of NC, PD, LOW, and HIGH groups in ELISA experiments. ns: no significance; *: *p* < 0.05; **: *p* < 0.01; ***: *p* < 0.001. (G): Analysis of urine exosome protein LCAT expression in diabetic patients with different fasting glucose levels. (a): Expression of NC group, Level 1 diabetic group, Level 2 diabetic group, and Level 3 diabetic group in mass spectrometry experiment; (b): Expression of NC, PD, Level 1 diabetic group, Level 2 diabetic group, and Level 3 diabetic group in ELISA experiments. ns: no significance; **: *p* < 0.01; ***: *p* < 0.001. (H): Evaluation of diagnostic efficacy of urine exosome protein LCAT. (a): ROC curve based on LC‐MS data; (b): ROC curve based on ELISA data. AUC, area under the curve; CI, confidence interval.

Twenty‐nine proteins were aggregated in the cholesterol metabolism pathway, and seven proteins were up‐regulated in the urine exosomes of diabetic patients, including LCAT, LPA, APOH, CETP, APOA2, APOA1 and APOA4. Among them, LCAT was the most significantly up‐regulated protein and was selected as target protein for further study (Figure 2A). GO analysis (Figure 2B) revealed that 29 proteins were primarily involved in cholesterol transport (BP), cholesterol binding (MF), and were localised to plasma lipoprotein particles and lipoprotein particles (CC). KEGG pathway analysis demonstrated significant enrichment in cholesterol metabolism pathways (Figure 2C).

#### 3.1.8 Expression Analysis of Cholesterol Metabolism‐Related Protein LCAT

Mass spectrometry showed that the expression of LCAT protein in urine exosomes of diabetic patients was higher than that of the NC group (Figure 2D‐a). ELISA showed that compared with the NC and PD groups, the expression of urine exosome protein LCAT in diabetic group increased (Figure 2D‐b). Western blots showed that the changes of LCAT protein in urine exosomes (Figure 2D‐c), urine (Figure 2E‐b) and blood (Figure 2E‐a) of diabetic patients were consistent, and the expression was significantly increased.

#### 3.1.9 Clinical Diagnostic Value of Urine Exosome Protein LCAT

According to the level of HbA1c in diabetic patients, the diabetic patients were divided into LOW group (< 8%) and HIGH group (≥ 8%) by HbA1c, and the urine exosome protein LCAT in these two groups was higher than that in the NC group (Figure 2F). Combined with the FBG index and two‐hour postprandial blood glucose index, the diabetic patients were divided into three grades: Level 1: < 6.1; Level 2: 6.1–7.8; Level 3: > 7.8 mmol/L, and the expression of LCAT protein in urine exosomes of diabetic patients exhibited an initial increase followed by a decreasing trend with disease progression (Figure 2G). Additionally, our analysis revealed no statistically significant differences in key renal function parameters (eGFR and creatinine) across all predefined subgroups of patients with diabetes (e.g., LOW/HIGH groups; Level 1/2/3 groups) (Supporting Information S4).

The ROC analysis of mass spectrometry data (Figure 2H‐a) revealed that urine exosome LCAT protein had an area under the curve (AUC) of 0.840 (95% CI: 0.727–0.954). Similarly, the ROC analysis of ELISA data (Figure 2H‐b) showed an AUC of 0.701 (95% CI: 0.574–0.821) for urine exosome LCAT protein.

### 3.2 Part II: Validation of Cellular and Mouse Models

#### 3.2.1 The Optimal Conditions for Establishing Insulin Resistance Cell Models

**FIGURE 3:**
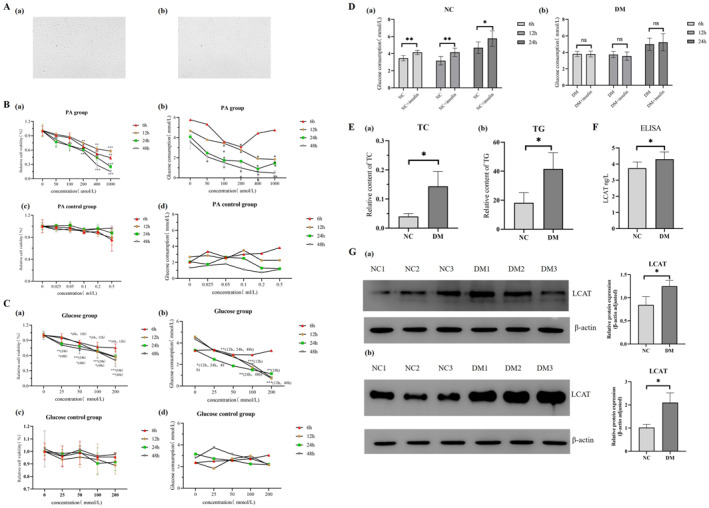
Construction of cell insulin resistance model and verification of target protein LCAT expression. (A): Morphology of normal BRL‐3A hepatocytes. (B): Effect of different concentrations of palmitic acid (PA) and ethanol on the relative survival rate and glucose consumption of BRL‐3A cells. (a): Relative cell viability changes after 6 h, 12 h, 24 h and 48 h of palmitic acid treatment; (b): Changes in glucose consumption after 6 h, 12 h, 24 h and 48 h of palmitic acid treatment; (c): Relative cell viability changes after 6, 12, 24, and 48 h of ethanol treatment; (d): Changes in glucose consumption after 6, 12, 24, and 48 h of ethanol treatment. *: *p* < 0.05; **: *p* < 0.01; ***: *p* < 0.001. (C): Effect of different concentrations of glucose and mannitol on relative survival and glucose consumption of BRL‐3A hepatocytes. (a): Relative cell viability changes after 6 h, 12 h, 24 h and 48 h of glucose treatment; (b): Changes in glucose consumption after 6 h, 12 h, 24 h and 48 h of glucose treatment; (c): Relative cell viability changes after 6 h, 12 h, 24 h and 48 h of mannitol treatment; (d): Changes in glucose consumption after 6, 12, 24, and 48 h of mannitol treatment. *: *p* < 0.05; **: *p* < 0.01; ***: *p* < 0.001. (D): Changes in glucose consumption after insulin stimulation in BRL‐3A hepatocyte insulin resistance model. (a): normal control group; (b): diabetic group. ns: no significance; *: *p* < 0.05; **: *p* < 0.01. (E): Expression changes of lipid metabolism markers in BRL‐3A hepatocyte insulin resistance model. (a): TC; (b): TG. *: *p* < 0.05. (F): Expression of cholesterol metabolism‐related protein LCAT in ELISA experiments. *: *p* < 0.05. (G): Expression of cholesterol metabolism‐related protein LCAT in Western blot experiments; *β*‐actin was used as the reference protein. *: *p* < 0.05.

BRL‐3A cells exhibited characteristic fibroblast‐like morphology (Figure 3A). Short‐term treatment with PA at 50 and 100 μmol/L for up to 12 h did not affect the cell survival rate (Figure 3B‐a,b). However, 100 μmol/L PA for 12h significantly reduced glucose consumption (*p* < 0.05). Ethanol controls showed no impact on survival or glucose uptake (Figure 3B‐c,d). In conclusion, the optimal concentration of PA is 100 μmol/L, and the optimal treatment time is 12 h.

The cell survival rate of BRL‐3A cells treated with 25 mmol/L glucose for 6 and 12 h showed no significantly difference compared to the normal control group (Figure 3C‐a,b). But glucose consumption began to decrease significantly (*p* < 0.05) when BRL‐3A cells were treated with 25 mmol/L glucose for 12 h. The mannitol osmotic control group demonstrated no significant differences compared with normal controls (Figure 3C‐c,d). In conclusion, the optimal glucose concentration is 25 mmol/L, and the optimal treatment time is 12 h.

#### 3.2.2 Defective Insulin‐Stimulated Glucose Metabolism in Insulin‐Resistant Cells

Glucose consumption was significantly increased by insulin stimulation in the NC group. The diabetic group showed no significant change in glucose uptake following insulin stimulation (*p* > 0.05), which remained unaltered after extended culture periods of 6 h, 12 h, and 24 h (Figure 3D).

#### 3.2.3 Changes of Lipid Metabolism in Insulin‐Resistant Cells

As shown in Figure 3E, the TG and TC levels of cells in the diabetic group were higher than those in the NC group (*p* < 0.05). The results suggested that the synthesis of TG and TC was significantly increased in diabetic cells.

#### 3.2.4 Expression Analysis of Protein LCAT in Insulin‐Resistant Cells

In the ELISA, LCAT expression was significantly elevated in the DM (*n* = 6) compared with the NC (*n* = 6) (Figure 3F). For Western blot (Figure 3G), LCAT expression was significantly higher in the DM compared to NC (*p* < 0.05), with *β*‐actin serving as the internal reference protein.

#### 3.2.5 Basic Information of Type 2 Diabetic Mice

**FIGURE 4:**
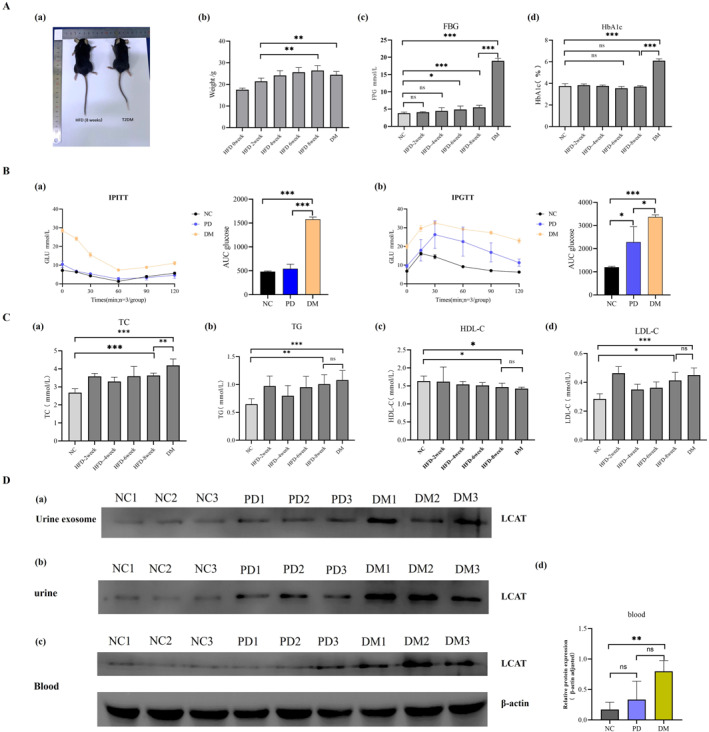
Construction of type 2 diabetic mouse model and verification of target protein LCAT expression. (A): Basic information of type 2 diabetic mice. (a): Representative morphological appearance of HFD for 8 weeks and diabetic mice; (b): Changes in body weight of mice fed with high‐fat diet for 8 weeks and diabetic mice; (c): Fasting blood glucose (FBG) levels of mice in each group; (d): Changes in glycosylated haemoglobin (HbA1c) levels of mice in each group. ns: no significance; *: *p* < 0.05; **: *p* < 0.01; ***: *p* < 0.001. (B): IPITT and IPGTT experiments in mice from NC, PD, and DM groups. (a): Changes in blood glucose levels in the IPITT experiment and area under the curve of the IPITT experiment; (b): Changes in blood glucose levels in the IPGTT experiment and the area under the curve of the IPGTT experiment. *: *p* < 0.05; ***: *p* < 0.001. (C): Trend changes of blood lipid metabolism index levels in NC group, PD group and DM group mice. (a): TC; (b): TG; (c): HDL‐C; (d): LDL‐C. ns: no significance; *: *p* < 0.05; **: *p* < 0.01; ***: *p* < 0.001. (D): Changes in the expression of urine exosomes, urine and blood cholesterol metabolism‐related protein LCAT of mice in NC group, PD group and DM group. (a): urine exosomes; (b): urine; (c): blood; (d): grey values measured using ImageJ software. ns, no significance; **: *p* < 0.01.

Throughout the type 2 diabetes mouse model establishment, NC mice exhibited good mental status and glossy fur. After 8 weeks of HFD feeding, both the prediabetic and diabetic groups showed significant weight gain with increased food and water consumption compared to the NC group. Following STZ injection, diabetic mice developed classic diabetic manifestations including polydipsia, polyphagia, polyuria and weight loss (Figure 4A‐a,b).

With prolonged HFD feeding, the mice exhibited progressively elevated blood glucose levels. With the injection of STZ, the blood glucose level and HbA1c level of diabetic mice increased significantly (Figure 4A‐c,d).

#### 3.2.6 Significantly Impaired Blood Glucose Regulation Capacity of Type 2 Diabetic Mice

IPITT results demonstrated that relative to both NC and PD groups, diabetic mice exhibited slower blood glucose reduction following insulin injection. At 120 min, diabetic mice maintained higher blood glucose levels with significantly greater AUC values versus controls. IPGTT analysis revealed elevated blood glucose levels in diabetic mice after glucose challenge compared to NC. Diabetic mice showed a rapid hyperglycaemic response that persisted at 120 min, failing to return to baseline levels, accompanied by increased AUC (Figure 4B).

#### 3.2.7 Trend of Blood Lipid Metabolism in Type 2 Diabetic Mice

Diabetic mice showed significantly elevated serum levels of TC, TG, and LDL‐C along with reduced HDL‐C compared to the NC group (Figure 4C). Notably, this change has been shown in prediabetic mice, suggesting that early‐stage lipid metabolic dysregulation precedes full diabetes development.

#### 3.2.8 Expression of Protein LCAT in Urine and Blood of Type 2 Diabetic Mice

The expression of LCAT in urine exosomes, urine and blood of diabetic mice was higher than that in the NC group (Figure 4D). This upregulated LCAT expression observed in murine models closely mirrored the observed patterns in human subjects.

#### 3.2.9 HE Staining of Tissues From Type 2 Diabetic Mice

**FIGURE 5:**
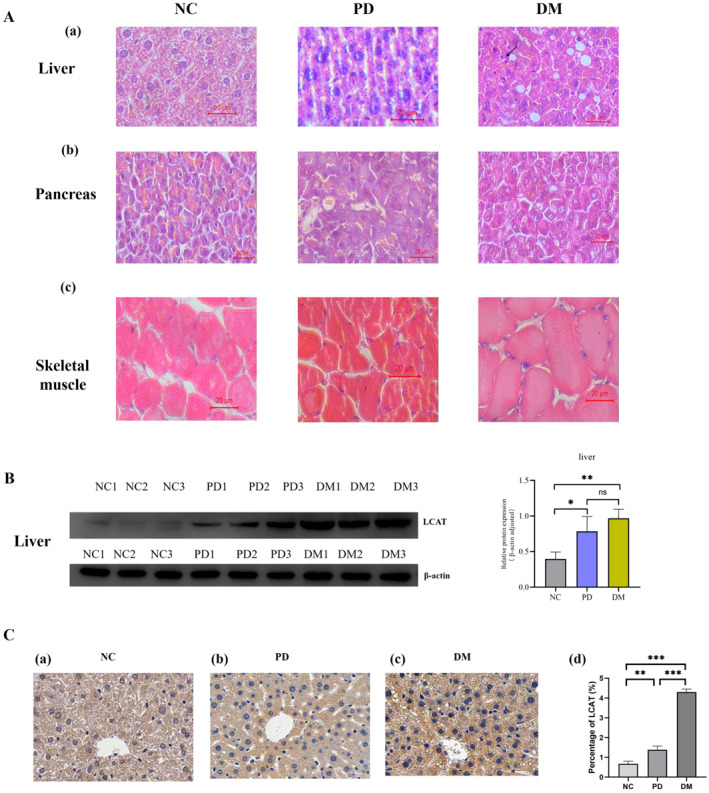
Analysis of tissue morphology and target protein LCAT expression in type 2 diabetic mice. (A): HE staining of tissues from mice in NC, PD, and DM groups. (a): liver; (b): pancreas; (c): skeletal muscle. (B): Changes in the expression of the target protein LCAT in the liver of mice in the NC, PD, and DM groups. *β*‐actin was used as an internal reference protein, and the grey value of LCAT expression was measured using ImageJ. ns, no significance; *: *p* < 0.05; **: *p* < 0.01. (C): Immunohistochemistry of liver tissue sections of mice in NC, PD, and DM groups. (a): The expression of LCAT in the liver of normal control mice; (b): The expression of LCAT in the liver of prediabetic mice; (c): The expression of LCAT in the liver of diabetic mice; (d): ImageJ measures the relative expression levels of LCAT in the three groups after adjustment. **: *p* < 0.01; ***: *p* < 0.001.

In liver tissue sections, the NC group exhibited regularly arranged, morphologically intact hepatocytes. In contrast, prediabetic mice showed hepatocyte disorganisation, whereas diabetic mice displayed marked hepatic steatosis characterised by disordered cell arrangement, cellular hypertrophy, and vacuolar degeneration. Compared with the NC group, diabetic mice had larger skeletal muscle cells, loose pancreatic tissue and enlarged histiocytes. Notably, pancreatic tissue alterations in diabetic mice were not as pronounced as those observed in the liver and skeletal muscle (Figure 5A).

#### 3.2.10 Expression of Protein LCAT in Liver of Type 2 Diabetic Mice

Western blot demonstrated elevated LCAT expression in the liver tissue of prediabetic and diabetic mice compared to the NC group (Figure 5B). Immunohistochemical analysis revealed significantly elevated LCAT protein expression in the liver tissues of diabetic mice, with prediabetic mice already demonstrating an initial upregulation of LCAT expression (Figure 5C).

## 4 Discussion

Disruption of lipid homoeostasis is closely related to diabetes pathogenesis. Logistic regression identified elevated TG and reduced HDL‐C as independent risk factors for the onset of diabetes, suggesting that lipid metabolism disorder is associated with the onset of diabetes [16, 17]. Notably, females exhibited higher TC and HDL‐C but lower TG levels than males in both NC and PD groups, a difference that may contribute to the higher prevalence of diabetes in males.

Twenty‐nine lipid metabolism proteins were identified in urine exosomes, among which lecithin cholesterol acyltransferase (LCAT) showed significant upregulation in diabetes compared to NC and was found to be associated with HDL; therefore, it was selected as the target protein. To validate the expression of LCAT, this study measured LCAT levels in urine, urine exosomes, and blood using MS, ELISA and Western blot. The results confirmed that LCAT exhibited systemic upregulation across biofluids under diabetic conditions.

The LCAT gene is located in human chromosome 16q22.1 [18], and its encoded protein LCAT serves as a central enzyme in the lipid metabolism network. LCAT is responsible for the generation of more than 90% of plasma cholesteryl ester [19], and it transports excess cholesterol from peripheral tissues to the liver by driving cholesterol reverse transport [18, 20]. By esterifying free cholesterol through its interaction with phosphatidylcholine, LCAT drives the conversion of small HDL precursors into mature spherical HDL particles [21]. This process significantly enhances the ability of HDL to remove cholesterol from peripheral tissues. Accumulating evidence indicates that LCAT overexpression is associated with the development of various malignancies, including colorectal and breast cancers [22, 23].

Compared with the normal control group, the diabetic model group exhibited elevated LCAT protein expression. This result demonstrates consistent trends with the LCAT alterations observed in clinical diabetes cases, providing further support for the compensatory upregulation of LCAT during insulin resistance.

In this study, a type 2 diabetic mouse model was established using the strategy of a ‘high‐fat diet combined with low‐dose STZ injection’ [24], which systematically simulated the pathological process of human diabetes from the compensated to the decompensated phase of glucose and lipid metabolism. TG, TC, and LDL‐C levels progressively increased, while HDL‐C levels gradually declined under HFD conditions. After the injection of STZ, lipid metabolism disorders were further aggravated, indicating that significant deviation in lipid metabolism indicators could be detected prior to the onset of diabetes, supporting the theoretical hypothesis of ‘lipotoxicity driving diabetes’ [25, 26]. Subsequent western blot revealed upregulated LCAT expression in urine, urine exosomes, and serum of diabetic mice. This trend closely matched the observations in clinical samples of diabetic patients, suggesting that LCAT may serve as a key regulatory molecule in metabolic compensation during diabetes.

Skeletal muscles play a critical role in insulin‐dependent glucose uptake and utilisation [27]. The liver is the primary site of LCAT synthesis and secretion [28]. Impaired insulin secretion by pancreatic *β* cells disrupts blood glucose homoeostasis [29]. Our histopathological analysis in mice revealed disorganised hepatocyte architecture at the early stage of diabetes, along with an increased proportion of vacuolar degeneration in the diabetic phase. In contrast, no significant structural damage was observed in the skeletal muscle or pancreas, implying that the liver may be the priority target organ for early pathological accumulation in diabetes. Hepatic LCAT was significantly increased in prediabetic mice relative to NC and maintained at high levels during the diabetic stage, indicating that dysregulated LCAT regulation may contribute to sustained metabolic dysfunction during diabetes progression.

Based on current literature, we hypothesise that during the prediabetic phase, hepatic LCAT is compensatorily upregulated in response to HDL‐C reduction caused by dyslipidemia. This adaptive mechanism likely aims to restore reverse cholesterol transport homoeostasis through enhanced HDL maturation, thereby delaying lipotoxic injury to pancreatic *β* cells. However, as diabetes progresses to the decompensated stage, persistent *β*‐cell apoptosis and insulin secretory failure lead to the collapse of this compensatory LCAT synthesis mechanism. Consequently, LCAT expression declines from its peak level, ultimately resulting in a dual deficiency of both HDL‐C and LCAT, which exacerbates systemic metabolic dysregulation.

Urine exosomes represent a promising source of novel biomarkers for diabetes [30]. The area under the ROC curve of the urine exosome protein LCAT was 0.840, indicating good diagnostic efficiency for diabetes. Moreover, LCAT expression exhibits a dynamic ‘compensation‐decompensation’ pattern during disease progression. In the early stage of diabetes, the expression of LCAT in urine exosomes is increased, but in the late stage of diabetes (defined as HbA1c ≥ 8% or FBG > 7.8 mmol/L), LCAT expression decreases relative to its peak, potentially reflecting the threshold of *β*‐cell failure and signalling disease progression. Given that in our cohort, there was no significant difference in glomerular filtration function between the diabetes groups (Supporting Information S4). In the late stage of diabetes, LCAT in urine exosomes is reduced, which may be due to the impairment of LCAT synthesis in the liver rather than the impairment of renal excretion caused by diabetes. In conclusion, this finding demonstrates that urine exosome LCAT holds significant application value for the non‐invasive diagnosis and disease progression monitoring of diabetes, and lays a foundation for the development of non‐invasive detection technology based on urine exosome LCAT.

## 5 Conclusions

Through integrated proteomic analysis combined with validation in cellular and animal models, this study systematically elucidates the characteristics of lipid metabolism disorders during the development and progression of diabetes. This study demonstrates that significant alterations in lipid metabolism markers are detectable even at the prediabetic clinical stage. Notably, as a key lipid metabolism regulator protein, LCAT exhibits significant compensatory upregulation during diabetes progression. Our proteomics analysis revealed characteristic dynamic changes in urine exosome LCAT, highlighting its potential as a novel molecular marker for early diabetes diagnosis and disease monitoring with promising clinical utility.t

## Author Contributions

M.Z. took charge of all the work and participated in its design. T.L. carried out most of the experiments and drafted the manuscript. N.L., Q.M. and T.L. did part of the experiments. All authors have read and approved the final manuscript.

## Funding

This work was supported by Validation and application development of a new urine diagnostic and monitoring marker test in type 2 diabetes‐related diseases (Z211100002921040), Capital Medical University Scientific Research Development Fund (PYZ24197) and the Beijing High‐Level Innovation and Entrepreneurship Talent Support Program (Young Top Talent Projects) (G202521124).

## Ethics Statement

This study was conducted in line with the principles of the Declaration of Helsinki. Approval was granted by the Ethics Committee of Beijing Shijitan Hospital, Capital Medical University (sjtkyll‐lx‐2022‐134). This animal experiment protocol was approved by the Experimental Animal Ethics Committee of Beijing Shijitan Hospital, Capital Medical University (sjtkyll‐lx‐2023‐073).

## Consent

Informed consent was obtained from all the individual participants included in the study.

## Conflicts of Interest

The authors declare no conflicts of interest.

## Supporting information


Supporting Information S1

Supporting Information S2

Supporting Information S3

Supporting Information S4

**Figure S1:** Clinical data analysis of diabetic patients. (A): Expression analysis of clinical lipid metabolism indicators in diabetic group (DM), prediabetic group (PD) and normal control group (NC) subjects. a: TC; b: TG; c: HDL‐C; d: LDL‐C. ns: no significance; ***:*p* < 0.001. (B): Gender differences in clinical lipid metabolism parameters among diabetes mellitus prediabetes and normal control subjects. a: normal control group; b: prediabetic group; c: diabetic group. ns: no significance; *: *p* < 0.05; **: *p* < 0.01; ***: *p* < 0.001.

## Data Availability

The data that support the findings of this study are available from the corresponding author upon reasonable request.
