# Supplementary material for: Evaluation of Urine Exosome Lecithin Cholesterol Acyltransferase as a Biomarker for Diabetes Diagnosis and Dyslipidemia
Source: Diabetes Metab Res Rev. 2026 Mar 3;42(3):e70133. doi: 10.1002/dmrr.70133 (PMC12956041; doi:10.1002/dmrr.70133)
Supplement: Supplementary file 1 — Supporting Information S1 [file DMRR-42-e70133-s004.docx]

**Exosome isolation, qualification and characterization**

In this study, differential centrifugation combined with size exclusion chromatography was used to separate urine exosomes. The specific process is as follows: The cell debris and apoptotic bodies in the urine were removed by low-speed centrifugation (1500 g, 10 min), and then the supernatant was collected after high-speed centrifugation (10,000 g, 30 min), followed by sterile filtration using 0.22 μm PVDF filter membrane. The filtrate obtained was transferred to a 100 kDa ultrafiltration centrifuge tube (Amicon Ultra-15, Merck UFC910008) for preconcentration. Exosomes were extracted by qEV10/35 size-exclusion chromatography (SEC), the eluate was collected, and finally concentrated twice through a 100 kDa ultrafiltration tube, packaged and stored at − 80 ° C.

This study was conducted in strict accordance with the minimum experimental standard guidelines (MISEV2018) published by the International Society for Extracellular Vesicles (ISEV). A multimodal technology platform was used to systematically characterize the isolated urine exosomes. Specifically: (1) Transmission electron microscope (TEM)：The samples were stained with 2% uranyl acetate, and the bilayer membrane structure of the vesicles was observed at 80,000× magnification. (2) Nanoparticle Tracking Analysis (NTA): The concentration and size distribution of exosomes were quantitatively detected. Each sample was repeated for 3 times, and the particle concentration (particles/mL) and main distribution peak were recorded. (3) Western Blot (WB), including positive markers CD9, CD63, TSG101 and Alix, and Calnexin was used as a negative control to verify the purity of exosomes.
