# Supplementary material for: Evaluation of Urine Exosome Lecithin Cholesterol Acyltransferase as a Biomarker for Diabetes Diagnosis and Dyslipidemia
Source: Diabetes Metab Res Rev. 2026 Mar 3;42(3):e70133. doi: 10.1002/dmrr.70133 (PMC12956041; doi:10.1002/dmrr.70133)
Supplement: Supplementary file 2 — Supporting Information S2 [file DMRR-42-e70133-s003.docx]

**The basic clinical data of diabetic group (DM), prediabetic group (PD) and normal control group (NC)**

| **Variables** | **NC group** | **PD group** | **DM group** | ***P*** |
| --- | --- | --- | --- | --- |
| Number | 261 | 225 | 137 |  |
| Age, years | 46.00（41.00~51.00） | 47.72±8.24 | 48.20±8.10 | 0.177 |
| Gender, male/female | 180/81 | 160/65 | 92/45 |  |
| ALT, U/L | 19.00（15.00~27.50） | 22.00（17.00~29.00） | 20.00（14.00~31.00） | 0.320 |
| AST, U/L | 19.00（16.00~23.00） | 19.00（16.00~24.00） | 17.00（14.00~24.00） | 0.676 |
| TBIL, μmol/L | 15.50（12.35~19.40） | 15.40±4.85 | 13.10（10.10~16.35） | ＜0.001 |
| TP, g/L | 75.97±3.51 | 76.14±3.75 | 67.05±6.81 | ＜0.001 |
| ALB, g/L | 46.02±2.25 | 45.85±2.13 | 38.78±3.74 | ＜0.001 |
| ALP, U/L | 68.00（58.00~81.50） | 73.04±17.87 | 69.00（58.00~86.50） | 0.554 |
| GGT, U/L | 24.00（17.00~38.50） | 24.00（17.00~39.00） | 25.00（18.00~38.00） | 0.085 |
| Urea, mmol/L | 4.77（3.94~5.62） | 4.96±1.10 | 5.35±1.61 | 0.023 |
| Cr, μmol/L | 74.00（64.00~81.00） | 73.60±13.17 | 65.00（54.00~76.00） | 0.284 |
| eGFR, ml/min/1.73m^2^ | 103.00（94.50~107.00） | 99.89±10.27 | 100.00（89.00~110.00） | 0.056 |
| UA, μmol/L | 360.03±85.93 | 363.62±81.54 | 339.37±82.68 | 0.020 |
| TC, mmol/L | 4.70±0.74 | 4.64±0.70 | 4.55±0.95 | 0.163 |
| TG, mmol/L | 1.15（0.81~1.73） | 1.19（0.89~1.76） | 1.60（1.17~2.41） | ＜0.001 |
| FPG, mmol/L | 4.88±0.41 | 5.10±0.47 | 7.84（6.73~10.68） | ＜0.001 |
| HDL-C, mmol/L | 1.25±0.26 | 1.16（1.03~1.36） | 1.02±0.23 | ＜0.001 |
| LDL-C, mmol/L | 2.96±0.62 | 2.97±0.61 | 3.08±0.79 | 0.201 |
| HbA1c, % | 5.40（5.30~5.50） | 5.80（5.70~6.00） | 8.20（7.25~9.90） | ＜0.001 |
| RBC, *10^12^/L | 4.90±0.46 | 4.92±0.49 | 4.64±0.48 | ＜0.001 |
| HGB, g/L | 152.00（138.00~161.00） | 149.00（137.00~157.50） | 144.00（132.00~152.00） | ＜0.001 |
| PLT, *10^9^/L | 236.97±58.19 | 247.41±53.10 | 219.00（185.00~253.00） | 0.002 |
| WBC, *10^9^/L | 5.86±1.32 | 6.38±1.39 | 6.72±1.87 | ＜0.001 |

Note: ALT: Aspartate Aminotransferase, AST: Aspartate Aminotransferase, TBIL: Total Bilirubin, TP: Total Protein, ALB: Albumin, ALP: Alkaline Phosphatase, GGT: Gamma-Glutamyl Transpeptidase, Cr: Creatinine, eGFR: Estimated Glomerular Filtration Rate, UA: Uric Acid, TC: Total Cholesterol, TG: Triglyceride, FPG: Fasting plasma glucose, HDL-C: High-Density Lipoprotein Cholesterol, LDL-C: Low-Density Lipoprotein Cholesterol, HbA1c: glycated hemoglobin, RBC: Red Blood Cell Count, HGB: Hemoglobin, PLT: Platelet Count, WBC: White Blood Cell Count.
