# Supplementary material for: Evaluation of Urine Exosome Lecithin Cholesterol Acyltransferase as a Biomarker for Diabetes Diagnosis and Dyslipidemia
Source: Diabetes Metab Res Rev. 2026 Mar 3;42(3):e70133. doi: 10.1002/dmrr.70133 (PMC12956041; doi:10.1002/dmrr.70133)
Supplement: Supplementary file 3 — Supporting Information S3 [file DMRR-42-e70133-s005.docx]

**Table 1 Univariate logistic regression analysis of the incidence of diabetes**

| **Variables** | **B** | **SE** | **Wald** | **OR** | ***P*** | **95%CI** |
| --- | --- | --- | --- | --- | --- | --- |
| TBIL | -0.072 | 0.020 | 12.625 | 0.931 | ＜0.001 | 0.894~0.968 |
| DBIL | -0.166 | 0.060 | 7.665 | 0.847 | 0.006 | 0.753~0.953 |
| IBIL | -0.098 | 0.028 | 12.762 | 0.906 | ＜0.001 | 0.859~0.957 |
| TP | -0.478 | 0.041 | 132.666 | 0.620 | ＜0.001 | 0.572~0.673 |
| ALB | -1.069 | 0.099 | 115.640 | 0.343 | ＜0.001 | 0.283~0.417 |
| Urea | 0.162 | 0.067 | 5.922 | 1.176 | 0.015 | 1.032~1.341 |
| Cr | -0.016 | 0.007 | 5.835 | 0.984 | 0.016 | 0.971~0.997 |
| eGFR | -0.015 | 0.007 | 5.358 | 0.985 | 0.021 | 0.972~0.998 |
| UA | -0.003 | 0.001 | 7.461 | 0.997 | 0.006 | 0.994~0.999 |
| TG | 0.525 | 0.099 | 28.177 | 1.691 | ＜0.001 | 1.393~2.053 |
| HDL-c | -4.192 | 0.539 | 60.510 | 0.015 | ＜0.001 | 0.005~0.043 |
| RBC | -1.213 | 0.221 | 30.134 | 0.297 | ＜0.001 | 0.193~0.458 |
| HGB | -0.030 | 0.006 | 21.979 | 0.971 | ＜0.001 | 0.958~0.983 |
| PLT | -0.005 | 0.002 | 8.291 | 0.995 | 0.004 | 0.991~0.998 |
| WBC | 0.264 | 0.063 | 17.257 | 1.302 | ＜0.001 | 1.149~1.474 |
| LY% | -0.041 | 0.012 | 10.811 | 0.960 | 0.001 | 0.937~0.984 |
| MO% | 0.290 | 0.073 | 15.944 | 1.337 | ＜0.001 | 1.159~1.541 |
| NE% | 0.033 | 0.012 | 7.579 | 1.033 | 0.006 | 1.009~1.058 |

**Table 2 Multivariate logistic regression analysis of the incidence of diabetes**

| **Variables** | **B** | **SE** | **Wald** | **OR** | ***P*** | **95%CI** |
| --- | --- | --- | --- | --- | --- | --- |
| ALB | -1.244 | 0.183 | 46.366 | 0.288 | ＜0.001 | 0.201~0.412 |
| Urea | 0.735 | 0.231 | 10.133 | 2.805 | 0.001 | 1.326~3.279 |
| TG | 0.765 | 0.290 | 6.966 | 2.149 | 0.008 | 1.218~3.793 |
| HDL | -5.069 | 1.381 | 13.478 | 0.006 | ＜0.001 | 0~0.094 |
| WBC | 0.499 | 0.189 | 6.950 | 1.647 | 0.008 | 1.137~2.387 |
| MO% | 0.518 | 0.223 | 5.404 | 1.679 | 0.020 | 1.085~2.598 |
