# Supplementary material for: Evaluation of Urine Exosome Lecithin Cholesterol Acyltransferase as a Biomarker for Diabetes Diagnosis and Dyslipidemia
Source: Diabetes Metab Res Rev. 2026 Mar 3;42(3):e70133. doi: 10.1002/dmrr.70133 (PMC12956041; doi:10.1002/dmrr.70133)

**Figure 1** Expression of eGFR and CRE between the Low and High Groups of Diabetes.


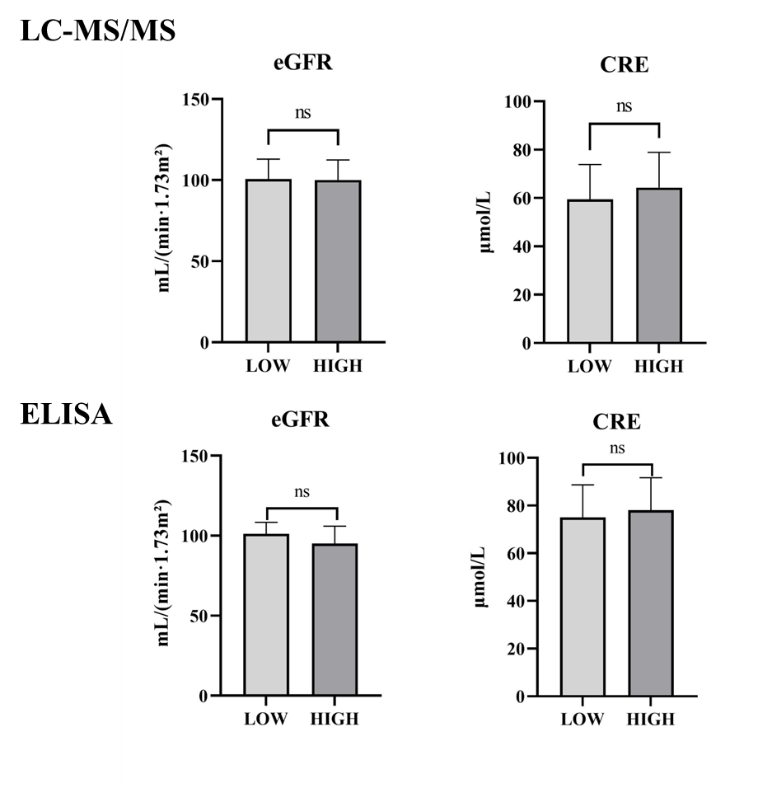


**Figure 2** Expression of eGFR and CRE among the Diabetes Level 1, Level 2, and Level 3 groups.


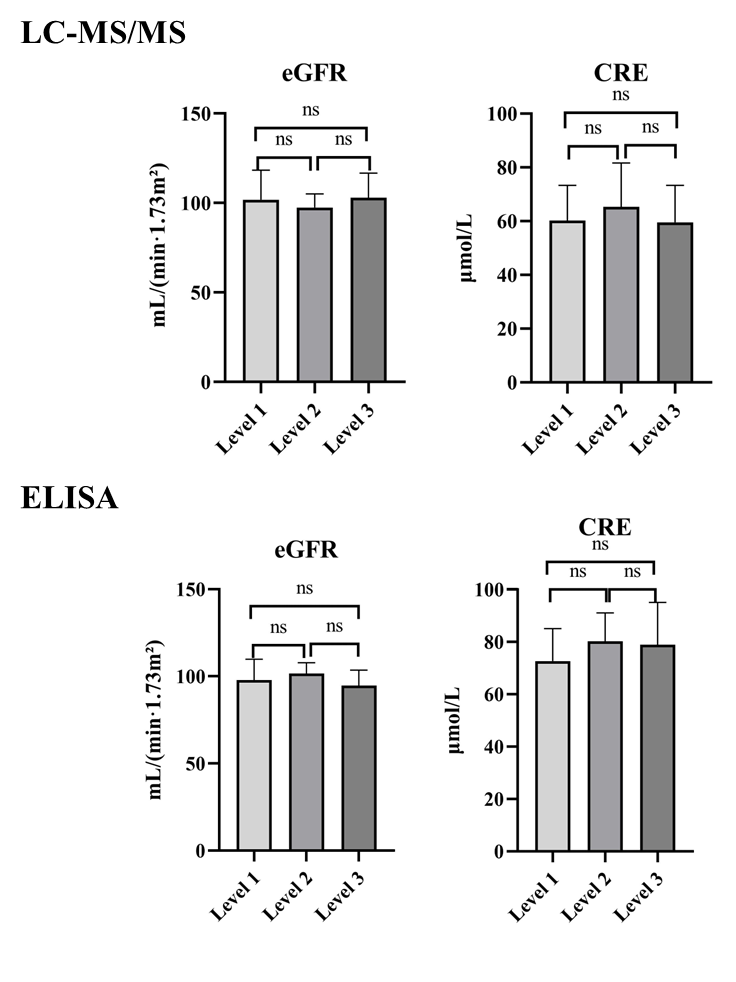

Supplement: Supplementary file 4 — Supporting Information S4 [file DMRR-42-e70133-s002.docx]
