# Supplementary material for: Evaluation of Urine Exosome Lecithin Cholesterol Acyltransferase as a Biomarker for Diabetes Diagnosis and Dyslipidemia
Source: Diabetes Metab Res Rev. 2026 Mar 3;42(3):e70133. doi: 10.1002/dmrr.70133 (PMC12956041; doi:10.1002/dmrr.70133)
Supplement: Supplementary file 5 — Figure S1: Clinical data analysis of diabetic patients. (A): Expression analysis of clinical lipid metabolism indicators in diabetic group (DM), prediabetic group (PD) and normal control group (NC) subjects. a: TC; b: TG; c: HDL‐C; d: LDL‐C. ns: no significance; ***:p < 0.001. (B): Gender differences in clinical lipid metabolism parameters among diabetes mellitus prediabetes and normal control subjects. a: normal control group; b: prediabetic group; c: diabetic group. ns: no significance; *: p < 0.05; **: p < 0.01; ***: p < 0.001. [file DMRR-42-e70133-s001.docx]

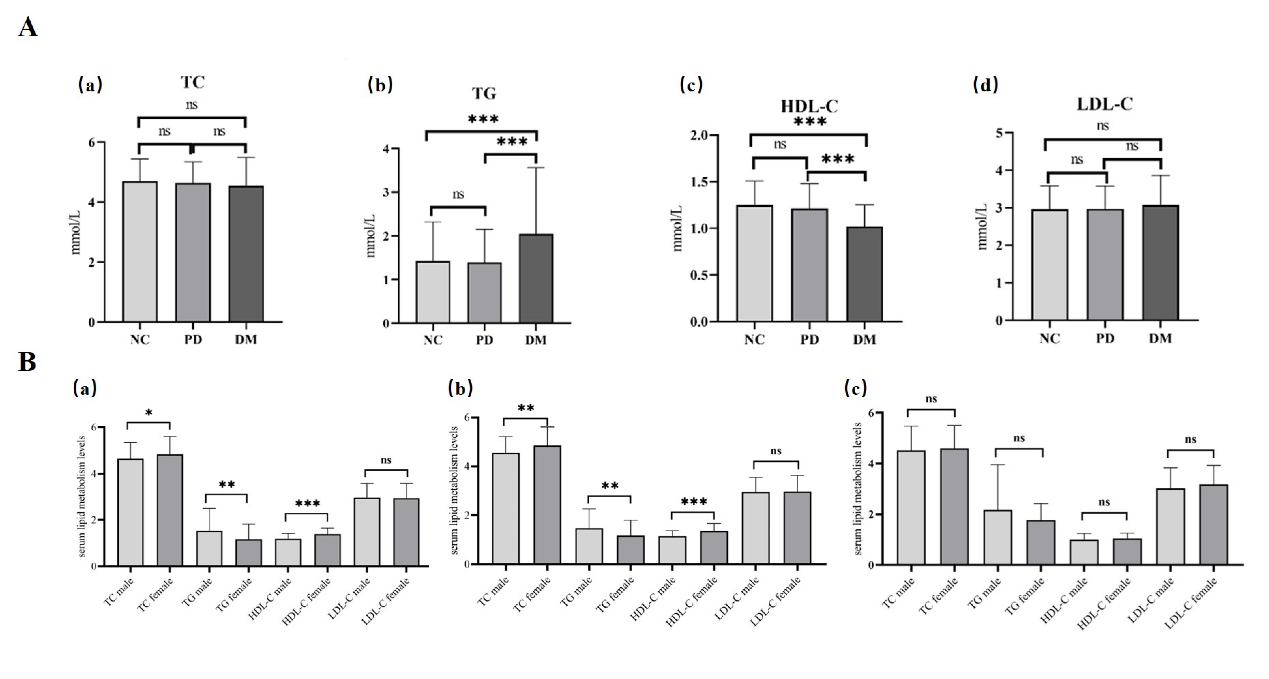


**Supplementary Figure 1. Clinical data analysis of diabetic patients**

A: Expression analysis of clinical lipid metabolism indicators in diabetic group (DM), prediabetic group (PD) and normal control group (NC) subjects. a: TC; b: TG; c: HDL-C; d: LDL-C. ns: no significance; ***: *P* < 0.001.

B: Gender difference analysis of clinical lipid metabolism indicators in diabetic group, prediabetic group and normal control group subjects. a: normal control group; b: prediabetic group; c: diabetic group. ns: no significance; *: *P* < 0.05; **: *P* < 0.01; ***: *P* < 0.001.
